# Prescreening of Mango *(Mangifera indica* L.) Leaves as a Potential Functional Food Ingredient: Techno-Functional and Antioxidative Characteristics

**DOI:** 10.3390/molecules30163381

**Published:** 2025-08-14

**Authors:** Génica Lawrence, Ingrid Marchaux, Ewa Pejcz, Agata Wojciechowicz-Budzisz, Remigiusz Olędzki, Adam Zając, Oliwia Paroń, Guylène Aurore, Joanna Harasym

**Affiliations:** 1 Campus de Fouillole, Université des Antilles, COVACHIM-M2E (EA 3592), UFR SEN, F-97110 Pointe-à-Pitre, France; genica.lawrence@univ-antilles.fr (G.L.); imarchaux@gmail.com (I.M.); guylene.aurore@univ-antilles.fr (G.A.); 2 Department of Biotechnology and Food Analysis, Wroclaw University of Economics and Business, Komandorska 118/120, 53-345 Wroclaw, Poland; agata.wojciechowicz-budzisz@ue.wroc.pl (A.W.-B.); remigiusz.oledzki@ue.wroc.pl (R.O.); oliwia.paron@ue.wroc.pl (O.P.); joanna.harasym@ue.wroc.pl (J.H.); 3 Department of Bioorganic Chemistry, Wroclaw University of Economics and Business, Komandorska 118/120, 53-345 Wroclaw, Poland; adam.zajac@ue.wroc.pl; 4 Adaptive Food Systems Accelerator-Science Centre, Wroclaw University of Economics and Business, Komadorska 118/120, 53-345 Wroclaw, Poland

**Keywords:** mango leaves, *Mangifera indica* L., functional properties, bioactive compounds, antioxidant activity, waste valorization

## Abstract

Mango (*Mangifera indica* L.) is cultivated in tropical and subtropical regions, with all parts of the tree—including leaves—used traditionally to treat diabetes, infections, pain, and other conditions. Mango leaves contain proteins, minerals, vitamins, and phenolic compounds, including mangiferin, quercetin, and kaempferol, whose content varies by cultivar. This study evaluated the functional and bioactive properties of dried mango leaves from five cultivars (Julie, DLO, Nam Dok Mai, Irwin, and Keïtt) to determine their potential for food and nutraceutical applications. Analyses included water- and oil-related parameters, swelling and solubility indices, foaming and emulsifying properties, and antioxidant activity (DPPH, ABTS, and FRAP in hydroalcoholic and water extracts), complemented by FT-IR/ATR spectroscopy. Significant differences between the five analyzed cultivars were observed. Irwin exhibited the highest antioxidant activity (2.65 ± 0.55 mg TE/g DM in DPPH assay), while Nam Dok Mai demonstrated superior foaming capacity (82.69 ± 7.79 mL). Strong correlations (r > 0.9) between reducing sugars and antioxidant capacity suggest cultivar selection based on sugar content could predict antioxidant potential. FT-IR confirmed the presence of polar phenolic and protein compounds. The results demonstrate that mango leaves offer cultivar-dependent functional and antioxidant attributes relevant to food systems. Their targeted valorization may support sustainable industrial applications and circular bioeconomy strategies, particularly in tropical regions where mango cultivation is widespread.

## 1. Introduction

Various plants and tree leaves can be considered for use in the food industry due to their nutritional quality, bioactive potential, and techno-functional properties. Mango (*Mangifera indica* L.) leaves (MLs) are a good example in numerous studies that have demonstrated a wide range of their phytochemicals and associated health benefits, where polyphenols are the predominant compounds. Among them, mangiferin, a xanthone glycoside known for its strong antioxidant activity, is particularly abundant in MLs [1,2]. Evidence also suggests its potential antidiabetic [3], antimicrobial, antispasmodic, antigenotoxic [4], and anti-breast cancer effects [5]. MLs contain phenolic acids, benzophenones, ascorbic acid, carotenoids, tocopherols, and other antioxidants such as flavonoids [4]. Specific flavonoids like quercetin and kaempferol have been linked to reduced oxidative stress, a key factor in the development of many chronic diseases. Additionally, leaf extracts appear to modulate blood glucose levels and improve insulin sensitivity, indicating potential in the management of type 2 diabetes [3,6]. MLs are also a notable source of protein [3], minerals (nitrogen, potassium, phosphorus, iron, sodium, calcium, magnesium), and vitamins A, B, E, and C. These leaves are already used as an alternative livestock feed in developing countries.

Although MLs have long been used in traditional medicine in China, India, and Africa—typically as dried leaves or powder for herbal teas—scientific interest in their application has grown in recent years, particularly in the food, pharmaceutical, and cosmetic sectors. A more detailed characterization of their chemical composition and safety remains necessary before broader integration into modern medicine [4] and functional foods. Beyond their nutritional and antioxidant properties, only limited studies to date have explored the techno-functional properties of MLs, such as water-holding capacity, emulsifying ability, and foaming capacity, for potential food applications. Understanding these functional properties is essential for their appropriate use in food formulations, particularly considering the increasing demand for sustainable ingredients. Furthermore, the food industry’s growing demand for natural antioxidants and functional ingredients, driven by consumer preference for clean-label products, creates opportunities for mango leaf valorization. The estimated 25–30% of mango tree biomass represented by leaves remains largely unutilized, representing a significant waste stream that could be converted into valuable functional ingredients.

In Guadeloupe, a French overseas region in the Caribbean, as many as 200 mango cultivars are grown. Previous studies have shown that mango fruit cultivars differ significantly in organoleptic, physicochemical, and nutritional properties. Preliminary results from our laboratory suggest that the composition of MLs—including physicochemical parameters, nutritional content, and antioxidant profile—also varies by cultivar.

This study was undertaken to comparatively evaluate the bioactive compound profiles and techno-functional properties of mango leaves from five selected cultivars grown in Guadeloupe. Although the antioxidant potential and phytochemical richness of mango leaves have been partially described in the literature, there is a noticeable lack of systematic studies addressing their functional attributes in relation to cultivar variability. It is hypothesized that the techno-functional properties and antioxidant potential of mango leaves are significantly influenced by varietal differences and that certain cultivars may offer superior profiles for targeted food applications. Furthermore, both aqueous and ethanolic extracts are expected to differ in their extractive efficiency and antioxidant activity, depending on the polarity and structure of the compounds involved. By bridging this knowledge gap, the present research not only expands the understanding of underutilized plant resources but also explores their potential valorization in functional food design and sustainable ingredient development. This work also aims to contribute to local agricultural diversification strategies and to foster circular bioeconomy initiatives in tropical regions such as Guadeloupe.

## 2. Results and Discussion

**Figure 1:**
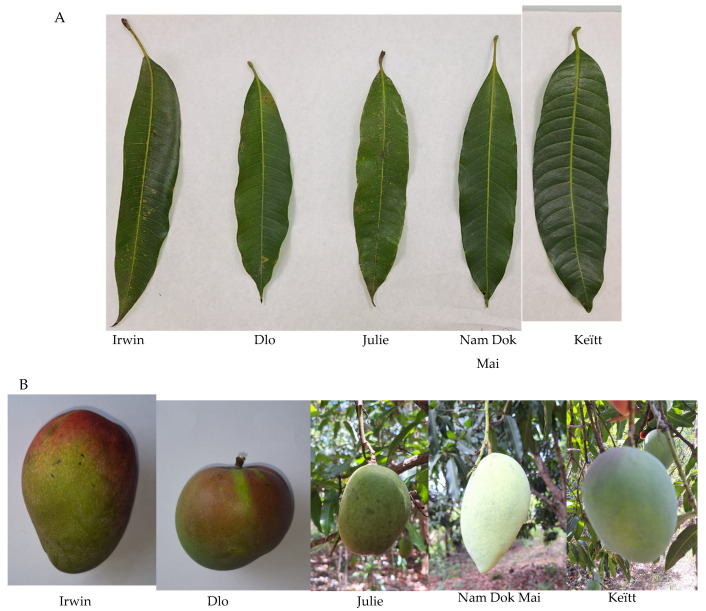
The mango leaves of different cultivars (**A**), Irwin, Dlo, Julie, Nam Dok Mai, and Keïtt, and the different forms of mango fruits associated with the mango leaves studied (**B**).

This study investigated the functional and bioactive properties of dried mango leaves from five cultivars (DLO, Nam Dok Mai, Irwin, Julie, and Keïtt) collected in Guadeloupe, France. After standardized drying and milling, the resulting powders were evaluated for their absorptional, emulsifying, foaming, and antioxidant properties. Representative images of the leaves and fruits are shown in Figure 1. Detailed procedures are described in Section 3.

### 2.1. Absorptional Characteristics of Mango Leaf Samples from Different Varieties

Understanding the water- and oil-binding properties of mango leaf powders is essential for determining their suitability in various food applications. These absorptional characteristics directly influence texture development, moisture retention, and processing behavior in food formulations. The evaluation encompassed both gravitational and mechanical water retention mechanisms, oil absorption capacity, and swelling behavior under thermal treatment conditions. Table 1 presents the absorptional characteristics of the five mango varieties, revealing distinct functional properties across water and oil-binding parameters.

The Nam Dok Mai variety demonstrated the highest water-holding capacity (WHC; 5.53 ± 0.14 g H_2_O/g DM) and the highest hydrophilic–lipophilic index (HLI; 1.576 ± 0.025), while exhibiting the lowest oil absorption capacity (OAC; 2.21 ± 0.08 g oil/g DM). The Julie variety showed the highest swelling power (SP; 4.72 ± 0.18 g H_2_O/g DM) and water absorption index (WAI; 5.14 ± 0.18 g H_2_O/g DM), along with the lowest water solubility index (WSI; 13.89 ± 0.95 g H_2_O/100 g DM) and the lowest HLI (1.307 ± 0.030). The Irwin variety exhibited the highest water absorption capacity (WAC; 3.76 ± 0.38 g H_2_O/g DM) and OAC (2.77 ± 0.13 g oil/g DM). The Keïtt variety presented the lowest WHC (4.64 ± 0.05 g H_2_O/g DM) but demonstrated the highest WSI (30.83 ± 6.47 g H_2_O/100 g DM), as well as the lowest WAI (3.60 ± 0.19 g H_2_O/g DM) and SP (2.67 ± 0.39 g H_2_O/g DM). The DLO variety displayed intermediate WHC (5.19 ± 0.33 g H_2_O/g DM) and moderate values across most absorption-related parameters.

Higher WAI values were observed (notably in Nam Dok Mai and Julie) than those reported by Kaur et al. [7], who obtained values around 3.0 g/g. This may suggest an increased water-binding capacity, potentially due to a greater number of hydrophilic groups or differences in the structural organization of polymers in the leaf matrix. Kaur et al. [7] also reported a WSI of 19.80%, while in the present study it ranged from 13.89 to 30.83 g H_2_O/100 g DM, indicating a possibly higher proportion of soluble compounds (e.g., polyphenols, sugars) in certain cultivars such as Keïtt and DLO. Furthermore, their reported OAC (3.67 g/g) exceeded the values observed here (maximum of 2.77 g oil/g DM), which may be attributed to differences in drying conditions, protein content, hydrophobic compound concentration, or particle size resulting from milling processes.

The variations in WAI, WSI, and WHC among the mango leaf powders may be partly explained by differences in surface and structural properties. As noted by Dey et al. [8], mango-leaf-derived biosorbents exhibited relatively high specific surface areas (36–43 m^2^/g) and abundant surface functional groups—such as hydroxyl, carboxyl, and phenolic moieties—which contribute to water retention and adsorption behavior. These features were also associated with enhanced performance in dye adsorption [9]. These findings support our results, where cultivars such as Nam Dok Mai and Julie exhibited the highest WHC and WAI, suggesting a more porous structure and a higher concentration of hydrophilic groups. Conversely, elevated WSI values observed in Keïtt and DLO may be linked to the increased solubility of low-molecular-weight components such as polyphenols and soluble fibers, which are known to be present in mango leaves and vary with genotype. These absorptional properties have direct implications for food applications: high WHC varieties like Nam Dok Mai (5.53 g H_2_O/g DM) would be suitable for moisture-sensitive products, while high OAC varieties like Irwin (2.77 g oil/g DM) could function as natural emulsifiers in lipid-rich formulations.

Overall, the absorption-related parameters show distinct varietal differences that may influence the suitability of each cultivar for food applications requiring specific hydration, oil interaction, or swelling behavior.

### 2.2. Foaming and Emulsification Characteristics of Mango Leaf Samples

Surface-active properties are critical for applications requiring foam generation or emulsion stabilization in food systems. The ability to create and maintain foams and emulsions determines the potential utility of mango leaf powders in aerated products, sauces, and other complex food matrices. Table 2 demonstrates the foaming and emulsification properties of the mango leaf varieties, revealing distinct surface-active characteristics across foam generation, stability, and emulsion formation parameters.

Nam Dok Mai variety exhibited the highest foaming capacity (82.69 ± 7.79 mL) and the highest foam stability (153.1 ± 14.4%), indicating strong ability to generate and maintain foam structures. Keïtt variety showed the highest emulsifying activity (3.83 ± 0.78%), while presenting moderate foaming capacity (67.29 ± 2.32 mL) and relatively high foam stability (129.4 ± 0.9%).

Irwin variety presented the lowest emulsifying activity (0.55 ± 0.00%) but demonstrated the highest emulsion stability (3.28 ± 0.00%), along with moderate foaming capacity (64.56 ± 1.55 mL) and foam stability (125.3 ± 1.3%).

Julie variety exhibited moderate foaming capacity (65.29 ± 9.76 mL), intermediate foam stability (121.9 ± 16.6%), moderate emulsifying activity (1.33 ± 0.37%), and relatively high emulsion stability (3.32 ± 1.31%). DLO variety displayed the lowest foaming capacity (59.79 ± 2.25 mL) and the lowest foam stability (112.8 ± 4.2%), while showing moderate emulsifying activity (1.33 ± 0.37%) and lower emulsion stability (2.22 ± 1.49%). The FC and FS values obtained in this study are notably higher than those reported by Kaur et al. [7].

The foaming and emulsification characteristics reveal significant varietal differences that influence the suitability of these mango leaf varieties for applications requiring specific surface-active properties, foam formation, or emulsion stabilization in food systems.

### 2.3. Bioactive Profiles of the Different Mango Leaf Varieties

The antioxidant potential of mango leaves represents a key factor in their valorization as functional food ingredients. Quantifying bioactive compounds and their antioxidant capacity across different cultivars enables the identification of varieties with superior health-promoting properties. Table 3 shows the bioactive profiles of the different mango varieties, revealing distinct antioxidant capacities and reducing sugar contents across both ethanol and water extraction systems.

The Irwin variety demonstrated the highest antioxidant activity across most assays, exhibiting the highest reducing sugar content (28.04 ± 2.46 mg GE/g DM), the highest DPPH activity in both ethanol (2.65 ± 0.55 mg TE/g DM) and water extracts (3.15 ± 0.65 mg TE/g DM), the highest ABTS activity in ethanol extracts (4.35 ± 0.85 mg TE/g DM) and water extracts (2.15 ± 0.35 mg TE/g DM), and the highest FRAP activity in water extracts (2.35 ± 0.28 mg FeSO_4_/g DM). The Keïtt variety showed strong antioxidant potential with high reducing sugar content (27.36 ± 0.77 mg GE/g DM) and substantial DPPH activity in water extracts (2.95 ± 0.48 mg TE/g DM), along with high ABTS activity in ethanol extracts (4.15 ± 0.68 mg TE/g DM). The Nam Dok Mai variety exhibited intermediate-to-high bioactive properties with moderate reducing sugar content (25.92 ± 0.37 mg GE/g DM) and good antioxidant activity across multiple assays, including DPPH water activity (2.89 ± 0.58 mg TE/g DM) and ABTS ethanol activity (4.12 ± 0.78 mg TE/g DM). DLO variety displayed moderate bioactive characteristics with reducing sugar content (24.67 ± 1.57 mg GE/g DM) and consistent antioxidant activities across both extraction systems. The Julie variety presented the lowest bioactive profile with the lowest reducing sugar content (20.61 ± 0.58 mg GE/g DM) and consistently lower antioxidant activities across all assays, including DPPH ethanol activity (1.95 ± 0.25 mg TE/g DM), ABTS water activity (1.65 ± 0.18 mg TE/g DM), and FRAP ethanol activity (1.65 ± 0.15 mg FeSO_4_/g DM).

The conducted studies have shown that the Julie mango leaves have the lowest content of reducing sugars (20.61 ± 0.58 mg GE/g DM), while the Irwin mango leaves have the highest content of reducing sugars among the analyzed samples (28.04 ± 2.46 mg GE/g DM). The values of total sugar content obtained by us are consistent with results of studies conducted on the leaves of *Mangifera indica* L. grown in Phagwara, Punjab region, India. These studies have shown that the total carbohydrate content in mango leaves can be as high as 49.76% of the powder mass derived from this raw material [7].

Sucrose (apart from glucose and fructose) is indicated to be the main form of non-structural carbohydrates present in mango leaves [10]. It is possible that during powder production, partial sucrose degradation occurred, leading to increased glucose and fructose levels [11]. It has also been confirmed that mango leaves contain arabinose and xylose, both reducing sugars from the aldopentose group, which may react with the Folin–Ciocalteu reagent [12].

The bioactive profiles demonstrate significant varietal differences in antioxidant capacity and reducing sugar content, indicating that the selection of mango leaf varieties should consider the intended application’s requirements for specific bioactive compounds and antioxidant properties. Therefore, due to the high sugar content, mango leaves (*Mangifera indica* L.) can be used in the production of bakery products, such as cookies and bread with desirable sensory qualities [13].

The observed antioxidant superiority of Irwin and Keïtt cultivars may reflect their genetic adaptation to environmental stressors. This suggests that leaf harvesting from stress-adapted cultivars could be strategically employed to maximize bioactive compound yields, supporting the development of standardized extraction protocols for commercial applications.

### 2.4. Pearson Correlation Analysis Between Functional and Bioactive Properties

The relationships between functional and bioactive properties enable rational cultivar selection and provide insights into the underlying mechanisms governing mango leaf functionality. Correlation statistical evaluation examined associations between all measured parameters to identify patterns that could inform practical decision making for ingredient selection (Table 4).

The Pearson correlation analysis revealed distinct correlation categories that provide comprehensive insights into the relationships between the functional properties and bioactive characteristics of the mango leaf varieties.

Antioxidant activity correlations demonstrated remarkably strong positive correlations (r ≥ 0.8) across all assays, indicating consistent antioxidant profiles across varieties and reliable measurement regardless of solvent or method employed. The analysis revealed that sugar content serves as the primary driver of antioxidant capacity, with correlation coefficients ranging from 0.940 to 0.984 between reducing sugars and various antioxidant parameters. This key finding demonstrates that varieties with high reducing sugar content, specifically Irwin and Keïtt, consistently show superior antioxidant activity across all assays.

The observed high antioxidant activity values of the Irwin mango leaf powder may be due to the adaptation of this variety to living in conditions of higher temperature and greater sunlight than those occurring on the islands of the Lesser Antilles archipelago, where temperatures average 28–30 °C. The ‘Irwin’ mango was developed in Florida as a cross between ‘Haden’ and ‘Lippens’. It has been reported that summers on the Florida peninsula are very warm and humid, with temperatures often exceeding 35 °C in the southern part of the peninsula [14]. This could make the ‘Irwin’ variety genetically better adapted to slightly higher temperatures and more sunlight (250–300 sunny days per year) than those prevailing on the island of Guadeloupe (180–240 sunny days per year) [15,16,17].

This may make the leaves of the ‘Irwin’ mango cultivar capable of producing higher amounts of antioxidants that counteract reactive oxygen species (ROS) formed as a result of the harmful effects of UV radiation on plant cells [18]. The results of the study confirm that exposure of young seed plants (developing from the embryo during germination), e.g., broad bean seedlings, to visible light or visible light combined with high-intensity UV-A and UV-C light causes a significant increase in the synthesis of polyphenolic compounds, including anthocyanins throughout the germination period. It was also confirmed that, as a result of intensive UV-A or UV-C radiation, there is a significant increase in the content of endogenous non-enzymatic antioxidants, such as ascorbate and glutathione (GSH) in cultivated broad bean seedlings [19]. Numerous studies have confirmed that increased synthesis of polyphenolic compounds, including flavonoids, reduces the damage caused by UV radiation by limiting the penetration of harmful ultraviolet (UV) radiation [20].

The ‘Julie’ mango variety comes from the islands of Trinidad and Jamaica and is very popular in cultivation in the Lesser Antilles [21]. It can be assumed that this variety shows a lower response to ultraviolet (UV) radiation from small-molecule (non-enzymatic) antioxidant systems. It has been confirmed that the leaves of many mango varieties are a rich source of highly polar antioxidants, such as mangiferin and benzophenone derivatives, such as iriflophenone 3-C-β-d-glucoside and iriflophenone 3-C-(2-O-p-hydroxybenzoyl)-β−d-glucoside, and phenolic acids, such as gallic acid [22].

This may explain the higher total antioxidant activities we obtained in aqueous extracts of powdered leaves of particular mango varieties than in ethanol extracts. It has also been confirmed that *Mangifera indica* leaves can be a rich source of another highly polar antioxidant compound—ascorbic acid (vitamin C). The content of ascorbic acid in the leaves of *Mangifera indica* grown in the Dhaka region of Bangladesh can be as high as 3.25 mg/g of dry leaves [23]. Ascorbic acid is able to react strongly with singlet oxygen, superoxide, and hydroxyl radicals [19]. Therefore, mango leaf powder has high potential to become a raw material used in the production of supplements and food products that can counteract oxidative stress in the human body.

The conducted studies showed that the highest reducing activity was characteristic of the Irwin mango leaves in an aqueous solution (2.35 mg FeSO_4_/g DM), and the lowest reducing activity was demonstrated by the Julie mango leaves in an ethanol solution (1.65 mg FeSO_4_/g DM). Moreover, the reducing activity of all tested mango leaf varieties was higher in an aqueous solution than in an ethanol solution. The high reducing activity of the analyzed mango leaf powders (measured in the FRAP test) observed in our study is, to a large extent, confirmation of their high activity in neutralizing reactive oxygen species.

The observed high capacity of Irwin mango leaves to reduce iron ions Fe^3+^ to Fe^2+^ may be due to the presence of certain minerals, such as potassium, phosphorus, iron, sodium, calcium, and magnesium ions in the tested raw material [24]. The presence of vitamin C and vitamin E (tocopherols and tocotrienols) has also been confirmed in mango leaves, which have high reducing properties [25].

The high reducing activity of Irwin mango leaves may also result from the presence of protein fractions that act as the building blocks of cells and play a key role in the construction of the photosynthetic apparatus and in cell and tissue signaling [26]. Studies have confirmed that the powder made from mango leaves contains a high amount of crude protein at 171.4 g/kg DM [4]. The presence of amino acids such as cysteine (Cys) and methionine (Met) in these proteins causes them to have high reducing properties [27]. This makes mango leaves a valuable raw material for the production of human food or protein feed for animals [28], which shows not only nutritional (building) properties but also strong reducing properties.

Functional property correlations exhibited several significant relationships, particularly among water-related properties. The analysis revealed a nearly perfect correlation between water absorption index and swelling power (r = +0.998), indicating that these parameters measure essentially the same underlying property. The water absorption index showed a strong negative correlation with the water solubility index (r = −0.922), demonstrating that high absorption capacity corresponds to low solubility, while the water solubility index negatively correlated with swelling power (r = −0.938), showing that high solubility reduces swelling capacity. The oil–water balance analysis revealed a very strong negative correlation between oil absorption capacity and the hydrophilic–lipophilic index (r = −0.942), indicating that high oil absorption corresponds to lower hydrophilicity. Moisture characteristics showed that water-holding capacity positively relates to water absorption index (r = +0.648), while displaying a complex negative relationship with water absorption capacity (r = −0.629).

Foaming correlations demonstrated exceptional predictability, with foaming capacity showing a nearly perfect correlation with foam stability (r = +0.990), indicating that foaming capacity serves as an excellent predictor of foam stability. The hydrophilic–lipophilic index strongly enhanced both foaming capacity (r = +0.847) and foam stability (r = +0.877), while oil absorption capacity negatively affected foaming ability (r = −0.916), demonstrating that oil absorption reduces foaming capacity.

Cross-functional relationships revealed important interactions between different property categories. Water absorption capacity showed strong positive correlations with antioxidant parameters (r = +0.601 to +0.791), indicating that water absorption enhances antioxidant extraction efficiency. Reducing sugar content emerged as a strong driver of antioxidant capacity across multiple assays. The relationship between foaming properties and other characteristics showed that oil absorption capacity maintains strong negative relationships with foaming parameters, while the hydrophilic–lipophilic index demonstrates strong positive relationships with foaming properties.

Key insights from the correlation analysis include five major findings. First, antioxidant synergy is evident as all antioxidant assays are highly intercorrelated (r > 0.9), suggesting that varieties can be reliably ranked for antioxidant potential using any single method. Second, the perfect correlation between water absorption index and swelling power (r = 0.998) indicates these parameters measure the same underlying property. Third, foaming predictability is exceptional, as foaming capacity perfectly predicts stability (r = 0.990), with both properties driven by hydrophilic characteristics. Fourth, an oil–water trade-off exists, as the strong negative correlation between oil absorption and hydrophilicity suggests that varieties optimize for either oil or water interactions. Fifth, sugar-driven antioxidants are evident, as reducing sugar content serves as the strongest predictor of antioxidant capacity across all methods.

Variety implications for practical applications indicate specific variety selections based on desired properties. For applications requiring high antioxidant activity, Irwin or Keïtt varieties should be chosen due to their high reducing sugar content correlating with superior antioxidant activity. For water-based applications, Julie or Nam Dok Mai varieties are optimal choices due to their high water absorption index and swelling power for applications requiring swelling properties. For foaming applications, the Nam Dok Mai variety represents the best choice with the highest foaming capacity and foam stability combined with optimal hydrophilic–lipophilic index. For oil-based applications, Irwin or DLO varieties are recommended due to their higher oil absorption capacity suitable for oil-based systems.

Emulsifying activity relationships demonstrated contrasting behaviors with water-related properties. WHC showed a strong negative correlation with emulsifying activity (r = −0.714), indicating that varieties with high water retention capacity exhibit reduced ability to form emulsions. This relationship suggests that strong water-binding properties may interfere with the protein–lipid interactions necessary for emulsion formation. Conversely, WSI displayed a strong positive correlation with emulsifying activity (r = +0.706), demonstrating that higher concentrations of soluble compounds enhance emulsification capacity. This finding indicates that water-soluble components, likely including soluble proteins and low-molecular-weight emulsifiers, contribute significantly to emulsion formation.

Emulsion stability correlations revealed a different characteristic compared to emulsifying activity. OAC showed a strong positive correlation with emulsion stability (r = +0.703), suggesting that varieties with higher lipid-binding capacity can better maintain emulsion structure over time. This relationship indicates that oil-binding properties are crucial for preventing phase separation in formed emulsions. WSI demonstrated a strong negative correlation with emulsion stability (r = −0.694), showing that high concentrations of soluble compounds, while beneficial for emulsion formation, may compromise long-term stability through increased mobility and potential phase separation.

The hydrophilic–lipophilic index showed a strong negative correlation with emulsion stability (r = −0.661), indicating that highly hydrophilic varieties produce less stable emulsions. This relationship suggests that balanced hydrophilic–lipophilic properties, rather than extreme hydrophilicity, are optimal for maintaining emulsion integrity.

### 2.5. Mid-Infrared (IR) Spectra of Mango Leaves

**Figure 2:**
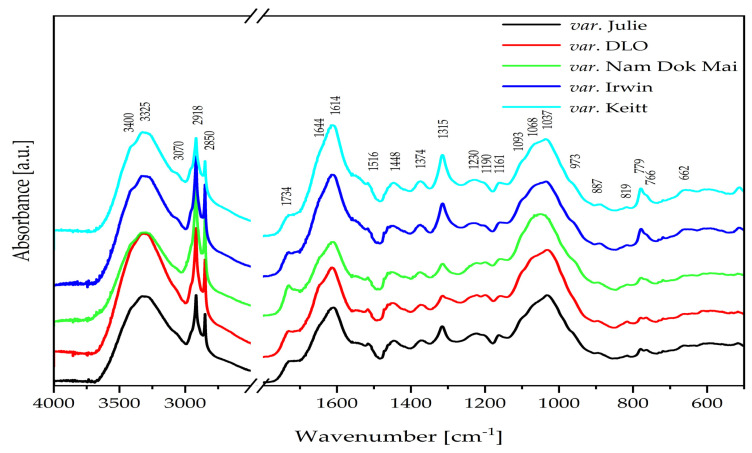
The FT-IR/ATR spectra of mango leaf samples from different varieties.

In the mid-infrared (IR) spectra of mango (*Mangifera* L.) leaves, a broad and intense absorption band is observed at ca. 3325 cm^−1^ (Figure 2), corresponding to υ(OH) vibrations [29].

The above band includes contributions from both free hydroxyl groups’ stretching vibrations υ(OH) and those involved in intra- and intermolecular hydrogen bonds (HBs). The band’s pronounced broadening results from an extensive hydrogen-bonding network, typical of plant matrices rich in polar compounds. The spectra reveal several distinct absorption regions, reflecting the complex molecular composition of the samples, with the band near 3325 cm^−1^ being particularly dominant [29]. In addition to phenolic compounds—such as flavonoids (e.g., quercetin) and gallic acid—sugars also play a significant role in this spectral region due to their numerous hydroxyl groups capable of bonding hydrogen. Their presence further enhances the intensity and width of the υ(OH) band, confirming a high content of polar, hydrophilic compounds in mango leaves, which is further supported by the appearance of a very strong absorption band at 1614 cm^−1^, which corresponds to the υ(C=C) vibrations in aromatic rings and conjugated C=O groups [29]. The band observed at 1516 cm^−1^ in the IR spectrum of mango leaves is primarily associated with δ(N-H) and υ(C–N) vibrations in amide groups (amide II), as well as υ(C=C) vibrations in the aromatic rings of flavonoids and other polyphenols. The presence of this band, together with the strong absorption at 1614 cm^−1^, corresponding to υ(C=C) and conjugated C=O groups, indicates a significant content of both polyphenolic compounds and proteinaceous substances with polar, hydrophilic characteristics [29]. These contributions enhance the intensity and broadening of the υ(OH) band around 3325 cm^−1^, confirming a high level of polar, hydrophilic matter in mango leaves. The vibration at 1644 can be attributed to the amide I (ν(C=O), δ(N-H)) bond from protein compounds [29,30,31,32,33].

The FT-IR spectrum of mango leaves, the bands at 2918 cm^−1^ and 2850 cm^−1^ (Figure 1), correspond to the stretching vibrations of methyl (ν_as_(CH)) and methylene (ν_s_(CH)) groups in the hydrocarbon chains of lipids and other organic compounds. These vibrations are characteristic of aliphatic alkyl groups commonly found in the cellular membrane structures and lipid components present in mango leaves. The FT-IR/ATR analysis suggests a low lipid content in the mango leaves. This is evidenced by sharp bands of medium/weak intensity at the following wavenumbers: 1734 (ν(C=O)), 1448 (δ(CH)/CH_2_), 1374 (δ(CH)/CH_3_), and 1230 and 1161 cm^−1^ (ν(C–O), δ(–CH_2_–)) [29,30,31,32,33,34].

The strong bands in the 1093–973 cm^−1^ (Figure 1) range of the IR spectrum of mango leaves are primarily associated with ν(C–O) and C–O–C vibrations characteristic of polysaccharides such as cellulose. Additionally, vibrations related to phenolic structures of lignin, particularly ether and aromatic bonds, may also be present in this region. The presence of these bands indicates a significant contribution of structural cell wall components, including cellulose and lignin, which determine the mechanical properties of mango leaves [29,30,31,32,33,35].

The band at 1315 cm^−1^ in the IR spectra of mango leaves is primarily attributed to a combination of δ(O–H) deformation vibration and ν(C–O) vibration associated with lignin, hemicellulose, and polyphenolic compounds. The band observed near 779 cm^−1^ corresponds to aromatic ring vibrations of lignin and polyphenols, as well as bending modes of polysaccharide carbohydrate rings. Notably, the intensity of these bands varies among mango cultivars, with the Irwin and Keïtt varieties exhibiting the strongest bands, indicating a higher content of lignin, structural polysaccharides, and polyphenols that contribute not only to the mechanical strength but also to the antioxidant activity of the leaves [29,30,31,32,33,35].

## 3. Materials and Methods

### 3.1. Materials

The mango leaves (ML) of five cultivars were used in this study: DLO, Nam Dok Mai, Irwin, Julie, and Keïtt. MLs were collected from identified specimens at ASSOFWI farm, located in Bouchu district at Vieux-Habitants (16°05′17.8″ N, 61°44′08.2″ W), in Guadeloupe island (French West Indies), in February 2024. ASSOFWI farm is an independent association under the law of 1901, whose activities are dedicated to education for the public and research. It has a collection of 6 mango cultivars. On the same day of collection, MLs were carefully washed with tap water to remove any soil, spots, or debris and then washed a second time with distilled water and dried slightly with a tissue. The mango leaves were then placed in a food dryer (Tauro Essiccatori Biosec Pro 2200 W—Tauro Essiccatori Srl, Vicenza, Italy) set to 42 °C and 30% relative humidity for 72 h. After drying, the leaves were ground to a fine powder using an electric grinder (Taurus Aromatic 150 W, Group Taurus, Barcelona, Spain). The resulting powders were immediately vacuum-packed and stored at room temperature (23 ± 1 °C) under optimal preservation conditions until experimental use (maximum 48 h). Each cultivar of mango leaf powder (MLP) was clearly identified and separately vacuum-packed.

### 3.2. Absorptional Characteristics of Mango Leaf Samples from Different Varieties

The absorptional properties of mango leaf powders from various cultivars were evaluated through a series of standardized procedures assessing water-holding capacity (WHC), water absorption capacity (WAC), oil absorption capacity (OAC), hydrophilic/lipophilic index (HLI), water absorption index (WAI), water solubility index (WSI), and swelling power (SP). All analyses were conducted in triplicate.

Water-holding capacity (WHC) and water absorption capacity (WAC) were determined according to procedures adapted from [36], with modifications distinguishing gravitational and mechanical water retention. For WHC, 3 g of dried mango leaf powder was placed in a pre-weighed centrifuge tube containing 30 mL of distilled water. The dispersion was left to sediment undisturbed at room temperature for 24 h. After this period, the supernatant was carefully decanted, and the remaining hydrated sediment was weighed. WHC was expressed as the grams of water retained per gram of dry sample, under gravitational force only. For WAC, 3 g of dried powder was dispersed in 30 mL of distilled water, vortexed for 30 s, and centrifuged at 3000× *g* for 10 min. The supernatant was discarded, and the residue was dried at 50 °C until a constant weight was obtained. WAC was calculated as the total mass of water absorbed and retained (g/g) after centrifugation and drying [36].

OAC was measured by mixing 3 g of sample with 30 mL of rapeseed oil under identical conditions as for WAC. After vortexing and centrifugation, the unbound oil was carefully removed, and residual oil was evaporated by placing the tubes in a drying oven (Vindon Scientific, Rochdale, UK) at 50 °C for 25 min. The OAC was calculated as grams of oil retained per gram of dry sample [36]. The HLI was calculated as the ratio of WAC to OAC, providing a comparative measure of the sample’s affinity for polar versus non-polar media.

For the determination of WAI, WSI, and SP, 3 g of mango leaf powder was suspended in 30 mL of distilled water in pre-weighed centrifuge tubes. The samples were heated in a water bath at 90 °C for 10 min (MLL147, AJL Electronics, Kraków, Poland) and then cooled to room temperature. Following centrifugation at 3000× *g* for 10 min (Thermo Fisher Scientific, Waltham, MA, USA), the supernatants were carefully decanted into pre-weighed Petri dishes and dried at 110 °C for 24 h in a laboratory oven (SML, Zalmed, Łomianki, Poland).

The sediment remaining in the centrifuge tubes was weighed to determine WAI, expressed as grams of water retained per gram of dry solids (g/g). WSI was calculated based on the mass of dried solubles in the supernatant and expressed as grams of solubles per 100 g of dry sample (g/100 g). Swelling power (SP) was determined as the ratio of the weight of the sediment to the dry matter content, expressed in g/g [37].

### 3.3. Foaming and Emulsification Characteristics of Mango Leaf Samples

The foaming and emulsifying properties of mango leaf powders were evaluated to assess their functionality in aerated and emulsion-based food systems. The following parameters were determined: foaming capacity (FC), foam stability (FS), emulsifying activity (EA), and emulsion stability (ES). All measurements were performed in triplicate.

To determine foaming properties, 1 g of mango leaf powder was dispersed in 50 mL of distilled water in a graduated 100 mL cylinder. The mixture was vigorously shaken by hand in a vertical motion for 5 min at room temperature. The volume of foam generated immediately after agitation was recorded and used to calculate FC (mL foam/g dry matter). The foam was then left undisturbed for 1 h, after which the foam volume was recorded again to determine FS, calculated as the percentage of the initial foam volume retained after 60 min [38].

EA and ES were measured following a modified centrifugation method. Briefly, 3 g of mango leaf powder was homogenized with 30 mL of distilled water and 30 mL of rapeseed oil for 1 min using a vortex mixer (Heidolph Reax, Schwabach, Germany). The emulsion was then centrifuged at 3000× *g* for 10 min (Thermo Fisher Scientific, Waltham, MA, USA), and the volume of the emulsified layer was measured. EA was expressed as the volume (mL) of the emulsified layer per total volume. For ES, the emulsion was heated to 70 °C for 30 min and then cooled to ambient temperature and left at rest for 1 h before remeasuring the emulsified layer. The stability was calculated as the percentage of emulsion volume retained after heat treatment and resting [39].

### 3.4. Bioactive Profiles of the Different Mango Leaf Varieties

#### 3.4.1. Extraction Procedure

Bioactive compounds were extracted using two solvent systems: distilled water and a hydroalcoholic mixture (ethanol/water, 80:20 *v*/*v*), each acidified with 1% HCl. The alcohol-to-water ratio used (80:20 *v*/*v*) was optimal in terms of the effectiveness and efficiency of the extraction process. The 1% addition of hydrochloric acid (HCl) to the extraction mixture was intended to facilitate the process of decomposition of the cell walls of the raw material used. Exactly 0.5 g of dried mango leaf powder was weighed and suspended in 10 mL of extraction solvent. The mixtures were vortexed for 1 min (MX-S, Chemland, Stargard, Poland) and agitated for 2 h at ambient temperature using a laboratory rotary shaker (MX-RD PRO, Chemland, Stargard, Poland). After incubation, samples were centrifuged at 3500× *g* for 10 min at 4 °C (MPW-350, MPW MED. INSTRUMENTS, Warsaw, Poland). The obtained supernatants were stored at 8 °C and analyzed within 24 h [40].

#### 3.4.2. Reducing Sugar Content

The reducing sugar content in the extracts was determined using a modified dinitrosalicylic acid (DNS) colorimetric method. A 0.5 mL aliquot of each extract was mixed with 0.25 mL of DNS reagent and heated in a boiling water bath for 5 min. After cooling to 50–60 °C, 3 mL of distilled water was added to dilute the reaction mixture. The absorbance was measured at 530 nm using a UV-Vis spectrophotometer (SEMCO S91E, EMCO, Sosnowiec, Poland). Glucose standards ranging from 100 to 800 μg/mL were used for calibration. The absorbance of a blank sample containing 0.5 mL of distilled water instead of the test extract was also measured. Results were expressed as milligrams of glucose equivalents (GEs) per gram of dry sample (mg GEs/g DM) [41].

#### 3.4.3. DPPH Radical Scavenging Activity

Antioxidant activity was assessed using the DPPH free radical scavenging assay. For each measurement, 0.0345 mL of the extract was added to 1 mL of a 0.1 mM methanolic DPPH solution. The mixture was incubated for 20 min in the dark at room temperature, and absorbance was measured at 517 nm. The absorbance of a blank sample was also measured, which contained 1 mL of DPPH working solution and 0.0345 mL of distilled water instead of the tested extract. A calibration curve was prepared using Trolox as the standard (100–800 μmol/L). The results were expressed as milligrams of Trolox equivalents (TE) per gram of dry sample (mg TE/g DM) [42].

#### 3.4.4. ABTS Radical Cation Decolorization Assay

The ABTS radical scavenging capacity was evaluated using a previously described method [40], with minor modifications. Briefly, 0.0204 mL of extract was added to 1 mL of a freshly prepared ABTS^•+^ solution, and the absorbance was measured at 734 nm precisely 10 s after mixing. The absorbance of a blank sample was measured, which contained 1 mL of ABTS working solution and 0.0204 mL of distilled water instead of the tested extract. Trolox was used as a standard (100–800 μmol/L), and antioxidant capacity was reported as mg TE/g DM [40].

#### 3.4.5. Ferric Reducing Antioxidant Power (FRAP) Assay

The FRAP assay was used to determine the electron-donating ability of the mango leaf extracts. A 0.0345 mL aliquot of each extract was mixed with 0.998 mL of freshly prepared FRAP reagent. After incubation for 15 min at 36 °C, absorbance was measured at 593 nm. The absorbance of a blank sample was measured, which contained 0.998 mL of FRAP working solution and 0.0345 mL of distilled water instead of the test extract. The results were calculated using a ferrous sulfate standard curve (100–800 μmol/L) and expressed as milligrams of FeSO_4_ equivalents per gram of dry sample (mg Fe^2+^/g DM) [40].

### 3.5. Mid-Infrared (IR) Spectra of Mango Leaves

The dried mango leaf powders (MLPs) were used directly for this experiment. FT-IR spectra were acquired using a Nicolet 6700 spectrophotometer (Thermo Fisher Scientific, Waltham, MA, USA) equipped with a diamond crystal in attenuated total reflectance (ATR) mode. Each sample was scanned 128 times in the mid-infrared region (4000–500 cm^−1^) at a resolution of 2 cm^−1^. The wavenumbers and corresponding band assignments for all samples are summarized in Table S1 (Supplementary Materials), including a legend of the spectroscopic abbreviations used. All measurements were performed in triplicate. Spectral data were processed and compared using OriginPro 2024b software (OriginLab Corporation, Northampton, MA, USA).

### 3.6. Statistical Analysis

All analyses were performed in triplicate, and the results were expressed as means ± standard deviations. One-way analysis of variance (ANOVA) was conducted to evaluate the effect of mango leaf variety on the studied functional and bioactive properties. Post hoc comparisons between group means were performed using Tukey’s honest significant difference (HSD) test at a significance level of *p* < 0.05. Pearson’s correlation coefficients (r) were calculated to assess relationships between selected functional parameters and antioxidant activities. All statistical analyses were carried out using Statgraphics Centurion XVII.I (StatPoint Technologies, Inc., Warrenton, VA, USA).

## 4. Conclusions

This study establishes clear cultivar-dependent functional and bioactive profiles for mango leaves, with practical implications for targeted applications. The identification of reducing sugar content as a reliable predictor of antioxidant capacity (r > 0.94) provides a simple screening tool for cultivar selection. Irwin and Keïtt cultivars emerge as premium antioxidant sources, while Nam Dok Mai offers superior foaming properties for aerated food systems.

The strong correlations identified enable rational cultivar selection: high-sugar cultivars for antioxidant applications, high-hydrophilicity cultivars for water-based systems, and specific cultivars for specialized functional requirements. These findings support the development of standardized processing protocols and quality specifications for mango leaf-derived ingredients.

However, successful commercialization will require addressing safety evaluation, sensory optimization, and regulatory compliance. The demonstrated variability underscores the critical importance of cultivar identification and botanical standardization in product development.

### Study Limitations and Future Directions

While this study provides valuable prescreening data, several limitations should be acknowledged. The analysis focused on dried leaves from a single geographic location and harvest time, which may not reflect seasonal or environmental variations. Future research should investigate the stability of these properties across different harvesting conditions and processing methods.

Additionally, safety evaluation, including anti-nutritional factors, sensory acceptability in food matrices, and the shelf-life stability of mango leaf ingredients, requires investigation. Scale-up feasibility and economic viability assessments are essential for commercial translation.

## Figures and Tables

**Table 1:** Absorptional characteristics of mango leaf samples from different varieties.

Sample	WHC [g H_2_O/g DM]	WAC [g H_2_O/g DM]	OAC [g oil/g DM]	HLI	WAI [g H_2_O/g DM]	WSI [g H_2_O/100 g DM]	SP [g H_2_O/g DM]
Julie	5.08 ± 0.10 ^b^	3.53 ± 0.15 ^a^^b^	2.70 ± 0.17 ^b^	1.307 ± 0.030 ^a^	5.14 ± 0.18 ^b^	13.89 ± 0.95 ^a^	4.72 ± 0.18 ^b^
DLO	5.19 ± 0.33 ^b^	3.59 ± 0.08 ^b^	2.69 ± 0.04 ^b^	1.336 ± 0.040 ^a^^b^	3.68 ± 0.34 ^a^	28.69 ± 7.72 ^c^	2.82 ± 0.56 ^a^
Nam Dok Mai	5.53 ± 0.14 c	3.48 ± 0.12 ^a^^b^	2.21 ± 0.08 ^a^	1.576 ± 0.025 ^c^	5.17 ± 0.29 ^b^	19.41 ± 2.70 ^b^	4.58 ± 0.33 ^b^
Irwin	5.19 ± 1.33 ^b^	3.76 ± 0.38 ^b^	2.77 ± 0.13 ^b^	1.354 ± 0.080 ^b^	4.72 ± 0.07 ^b^	15.68 ± 1.26 ^a^^b^	4.10 ± 0.10 ^b^
Keïtt	4.64 ± 0.05 a	3.73 ± 0.12 ^b^	2.49 ± 0.08 ^a^^b^	1.501 ± 0.088 ^b^^c^	3.60 ± 0.19 ^a^	30.83 ± 6.47 ^c^	2.67 ± 0.39 ^a^

WHC—water-holding capacity; WAC—water absorption capacity; OAC—oil absorption capacity; HLI—hydrophilic–lipophilic index; WAI—water absorption index; WSI—water solubility index; SP—swelling power; DM—dry matter. Lowercase letters mean significant differences in columns at *p* = 0.05.

**Table 2:** Foaming and emulsification characteristics of mango leaf samples from different varieties.

Sample	FC [mL]	FS [%]	EA [%]	ES [%]
Julie	65.29 ± 9.76 ^a^	121.9 ± 16.6 ^b^	1.33 ± 0.37 ^a^^b^	3.32 ± 1.31 ^b^
DLO	59.79 ± 2.25 ^a^	112.8 ± 4.2 ^a^	1.33 ± 0.37 ^a^^b^	2.22 ± 1.49 ^a^
Nam Dok Mai	82.69 ± 7.79 ^b^	153.1 ± 14.4 ^c^	1.66 ± 0.78 ^b^	2.10 ± 1.40 ^a^
Irwin	64.56 ± 1.55 ^a^	125.3 ± 1.3 ^b^	0.55 ± 0.00 ^a^	3.28 ± 0.00 ^b^
Keïtt	67.29 ± 2.32 ^a^	129.4 ± 0.9 ^b^	3.83 ± 0.78 ^c^	2.47 ± 0.39 ^a^

FC—foaming capacity; FS—foaming stability; EA—emulsifying activity; ES—emulsion stability. Lowercase letters mean significant differences in columns at *p* = 0.05.

**Table 3:** Antioxidant activities and reducing sugar contents of mango leaf powders extracted in ethanol and water systems.

Sample	Reducing Sugars	DPPH ETOH	DPPH H_2_O	ABTS ETOH	ABTS H_2_O	FRAP ETOH	FRAP H_2_O
	[GE mg/g DM]	[TE mg/g DM]				[FeSO_4_ mg/g DM]	
Julie	20.61 ± 0.58 ^a^	1.95 ± 0.25 ^a^	2.25 ± 0.35 ^a^	3.45 ± 0.45 ^a^	1.65 ± 0.18 ^a^	1.65 ± 0.15 ^a^	1.95 ± 0.12 ^a^
DLO	24.67 ± 1.57 ^b^^c^	1.85 ± 0.35 ^a^	2.45 ± 0.42 ^a^^b^	3.85 ± 0.65 ^b^	1.85 ± 0.28 ^a^^b^	1.78 ± 0.18 ^a^	2.05 ± 0.18 ^a^^b^
Nam Dok Mai	25.92 ± 0.37 ^c^	2.15 ± 0.45 ^a^	2.89 ± 0.58 ^b^	4.12 ± 0.78 ^b^	2.05 ± 0.15 ^b^	1.95 ± 0.12 ^a^	2.18 ± 0.25 ^b^
Irwin	28.04 ± 2.46 ^d^	2.65 ± 0.55 ^b^	3.15 ± 0.65 ^c^	4.35 ± 0.85 ^c^	2.15 ± 0.35 ^c^	2.05 ± 0.22 ^b^	2.35 ± 0.28 ^c^
Keïtt	27.36 ± 0.77 ^c^^d^	2.45 ± 0.35 ^b^	2.95 ± 0.48 ^b^^c^	4.15 ± 0.68 ^b^	1.95 ± 0.25 ^b^	1.85 ± 0.18 ^a^	2.25 ± 0.22 ^b^^c^

ETOH—ethanol extract; H_2_O—water extract. Lowercase letters mean significant differences in columns at *p* = 0.05.

**Table 4:** Pearson correlation coefficients between functional properties and antioxidant activities of mango leaf powders (*Mangifera indica* L.).

	WHC	WAC	OAC	HLI	WAI	WSI	SP	FC	FS	EA	ES	DPPH_H_2_O	ABTS_H_2_O	FRAP_H_2_O
WHC	1.000	−0.629	−0.471	0.486	0.648	−0.515	0.626	0.378	0.537	−0.714	−0.204	0.495	0.528	0.527
WAC		1.000	0.213	−0.137	−0.271	0.447	−0.291	−0.043	−0.191	0.204	0.343	0.612	0.367	0.717
OAC			1.000	−0.942	0.348	0.120	0.368	−0.916	−0.898	−0.425	0.703	0.425	0.155	0.332
HLI				1.000	−0.449	−0.253	−0.465	0.847	0.877	0.545	−0.661	−0.384	−0.094	−0.281
WAI					1.000	−0.922	0.998	0.195	0.360	−0.556	0.399	0.527	0.588	0.580
WSI						1.000	−0.938	−0.357	−0.487	0.706	−0.694	−0.419	−0.484	−0.442
SP							1.000	0.229	0.393	−0.573	0.443	0.548	0.608	0.598
FC								1.000	0.990	0.137	−0.413	−0.217	0.003	−0.128
FS									1.000	0.173	−0.381	−0.165	0.055	−0.072
EA										1.000	−0.409	0.140	−0.086	0.117
ES											1.000	−0.075	−0.188	−0.001
DPPH_H_2_O												1.000	0.949	0.989
ABTS_H_2_O													1.000	0.930
FRAP_H_2_O														1.000

WHC: water-holding capacity; WAC: water absorption capacity; OAC: oil absorption capacity; HLI: hydrophilic–lipophilic index; WAI: water absorption index; WSI: water solubility index; SP: swelling power; FC: foaming capacity; FS: foam stability; DPPH_H_2_O, ABTS_H_2_O, FRAP_H_2_O: antioxidant activities in water extracts.

## Data Availability

The original contributions presented in this study are included in the article. Further inquiries can be directed to the corresponding authors.

## Supplementary Materials

The following supporting information can be downloaded at https://www.mdpi.com/article/10.3390/molecules30163381/s1. Table S1. Wavenumbers of the bands observed in the IR spectra of mango leaf samples from different varieties, together with the proposed assignments of the bands.
