# Supplementary material for: Prescreening of Mango (Mangifera indica L.) Leaves as a Potential Functional Food Ingredient: Techno-Functional and Antioxidative Characteristics
Source: Molecules. 2025 Aug 14;30(16):3381. doi: 10.3390/molecules30163381 (PMC12388363; doi:10.3390/molecules30163381)
Supplement: Supplementary file 1 [file molecules-30-03381-s001.zip › molecules-3791362-supplementary.pdf]

Table S1. Wavenumbers of the bands observed in the IR spectra of mango leaf samples from different varieties, together with the proposed assignments of the bands

FT-IR/ATR peak position (cm^{-1})	Assignments
3700 – 3020s	ν(OH)HB, ν(NH)HB
2918s<br>2850m	ν(CH_2,CH_3)
1734w<br>1670sh	ν(C=O)
1644sh	Amide I
1614vs	υ(C=C) Ar ring
1545sh<br>1516m	δ(NH) Amide II, δ(CNH) amide, δ(NH) amine<br>δ(CNH) amide
1448m<br>1374m	δ(CH_2, CH_3) lipids and R side peptide chain
1315s	δ(O–H), ν(C–O)
1230m<br>1161w	(ν(C–O), δ(–CH_2–))
1093–973s	δ(ϕ) Phe ring
887w	ω(CH_2), γ(NH)
779w	(C=C) Ar ring
766w	
662w	

Abbreviations used: s, strong; m, medium; w, weak; v, very; sh, shoulder; ϕ, pyranoid ring; ν, stretching; δ, in-plane bending vibrations; γ, ω, out-of plane bendig.
